# Nematode Parasites in Baltic Sea Mammals, Grey Seal (*Halichoerus grypus* (Fabricius, 1791)) and Harbour Porpoise (*Phocoena phocoena* (L.)), from the German Coast

**DOI:** 10.1007/s11686-020-00246-7

**Published:** 2020-07-08

**Authors:** Michael Gabel, Stefan Theisen, Harry Wilhelm Palm, Michael Dähne, Patrick Unger

**Affiliations:** 1 Aquaculture and Sea-Ranching, Faculty of Agricultural and Environmental Sciences, University of Rostock, Justus-von-Liebig-Weg 6, 18059 Rostock, Germany; 2 German Oceanographic Museum Foundation, Katharinenberg 14-20, 18439 Stralsund, Germany

**Keywords:** Endoparasites, Molecular identification, C*ontracaecum osculatum*, *Pseudalius inflexus*, *Stenurus minor*

## Abstract

**Purpose:**

Endoparasitic nematodes of six harbour porpoises *Phocoena phocoena* and four grey seals *Halichoerus grypus*, stranded at the eastern coast of the Baltic Sea in Germany in winter 2019, were analysed in order to identify nematode parasites and to compare with recent studies from the same area.

**Methods:**

Endoparasitic nematodes were identified by using both morphological and molecular characters. The successfully obtained sequences of the rDNA marker regions ITS-1, 5.8S, ITS-2 from 29 anisakid and the rDNA marker region ITS-2 of 11 pseudalid nematodes were amplified.

**Results:**

Analyses revealed the presence of three parasite species, the anisakid nematode *Contracaecum osculatum* from grey seals and the pseudalid nematodes *Pseudalius inflexus* and *Stenurus minor* from the harbour porpoises. Other anisakid nematodes regularly occurring in the Baltic Sea, e.g. *Anisakis simplex* or *Pseudoterranova decipiens*, were not found.

**Conclusions:**

The prevalence of 100% and a severe parasite load in grey seals demonstrated a very high *C. osculatum* infection of Baltic Sea fish as their regular prey. Prevalence of 33% for parasites in harbour porpoises and minor infection rates, combined with a distinct lack of anisakid nematodes, are typical for the current situation of the porpoise parasite fauna in the Baltic Sea. Invasive parasite species as possible indicators for climate change could not be detected.

## Introduction

The Baltic Sea, a geologically young habitat [1], is inhabited by four species of marine mammals, not including vagrant species. Three of them are pinnipeds, the ringed (*Pusa hispida* (Schreber, 1775)), harbour (*Phoca vitulina,* L.) and grey seal (*Halichoerus grypus* (Fabricius, 1791)). The only cetacean reproducing inside the Baltic Sea is the harbour porpoise (*Phocoena phocoena* (L.)). Due to their adaptation to brackish water conditions and at least partly because of their independent, isolated reproduction in the Gulf of Bothnia and Gulf of Finland, the ringed and grey seal are treated as separate subspecies (*P.h. botnica* and *H.g. gryps)* [2]. In addition, morphological differences can also be observed [3].

The grey seal is the largest marine mammal living in the Baltic Sea. *Halichoerus grypus* can reach 165 to 210 cm in length and 250 kg in weight. A distinct sexual dimorphism can be observed, with males being much larger and bulkier, and showing a different head shape. The species was heavily reduced by extermination efforts in the 20^th^ century, organochlorine contamination and human fishing activities (drowning in fishing gear and fish community changes) [3]. Since the 1970s, the population is recovering at a growth rate of 6–10% per year. In the southern Baltic Sea growth rates currently reached approximately 20%. The species shows a broad prey spectrum, including other marine mammals, while the diet in the Baltic Sea is dominated by only a few fish species [4–7]. Their diet also changes with age [8, 9].

Anisakid nematodes (Anisakidae) (*Anisakis simplex* (Rudolphi, 1809)*, Pseudoterranova decipiens* (Krabbe, 1878) and *Contracaecum osculatum* (Rudolphi, 1802)), acanthocephalans of the genus *Corynosoma*, tapeworms, digeneans and, especially in pups, metastrongyloid nematodes (Metastrongylidae) (lungworms) are regular parasites of grey seals [10–12]. Co-infections are commonly observed. The parasite communities, however, change with age, most likely by a shift in diet or an improved immune system, reducing lungworms that are a frequent cause of death in their early life [10]. Anisakid nematodes are predominately represented by *C. osculatum* with about 60% of all recorded specimens, followed by *P. decipiens* (31%) and only a diminutive number of *A. simplex* (< 1%) [13, 14]. Almost all adult grey seals are heavily infected with *C. osculatum* (up to several hundred worms) [11]. The parasites life cycles benefit from growing populations of grey seals potentially causing higher parasite loads and abundances in Baltic Sea fish [15]. High parasite loads and abundances have been recorded in cods (*Gadus morhua*), which is beneath herring, whiting and roach a preferred prey of Baltic grey seals [9].

The harbour porpoise, the only native cetacean in the Baltic Sea, reaches a length of up to 180 cm and 80 kg. Sexual dimorphism in *Phocoena phocoena* is inconspicuous. Studies suggest three genetically and morphologically distinct populations in the Baltic Sea, with two being limited to the Baltic Proper and Danish waters [16]. The sub-population of the Baltic Proper is critically endangered [17]. Both populations were reduced by human activities in the 20^th^ century and may take a long time to fully recover. The populations show a distinct seasonal migration, moving from the Belt Sea south during summer down to north of Rügen, while retracting in winter up to the border between the Kattegat and Skagerrak [16]. Baltic proper porpoises seem to concentrate on Hoburgsbanken and Midsjöbanken in Sweden and Poland in summer during reproduction, while dispersing throughout the Baltic during winter [17]. The species is known to migrate through all regions of the Baltic Sea. Its diet shows seasonal variations, but mainly consists of gadids, herrings and to a lesser degree of gobies [4, 18, 19].

Parasites of the harbour porpoise in the Baltic Sea and adjacent waters are anisakid, metastrongylid and pseudalid (Pseudaliidae) nematodes, trematodes and tapeworms [20–22]. So far recorded pseudalid nematodes are *Pseudalius inflexus* (Rudolphi, 1808), *Torynurus convolutus* (Kühn, 1829), *Halocercus invaginatus* (Quekett, 1841) and *Stenurus minor* (Kuhn, 1829). They are settling in different parts of the respiratory system, with *S. minor* also invading the inner ear. Infections can result in lung oedema and pneumonia, as well as impaired hearing by haemorrhage and blockage of ear canals in case of heavy *S. minor* infection [22]. Infection rates can be high, with coinfections being common, but parasite loads are generally increasing with age [23]. Of the anisakid nematodes, *A. simplex* regularly infects the harbour porpoise while *C. osculatum* has rarely been reported [24, 25], but not from the Baltic Sea. The raphidascaridid nematode *Hysterothylacium aduncum* (Rudolphi, 1802) has been reported at high abundance and prevalence, but can be considered a relic (remaining undigested unlike its fish hosts) [26]. Infection rates with *Anisakis* range from 10-26% and can cause severe chronic ulcerative oesophagitis and gastritis when embedding in the intestinal walls [20]. Intradermal infections, resulting in ulcerative and granulomatous dermatitis are also described [27]. The trematode species *Campula oblonga* (Cobbold, 1858) and *Pholeter gastrophilus* (Kossack, 1910) have been also recorded in previous studies [21].

Based on extensive molecular analysis, this study aims to continue the ongoing research on nematode parasites of Baltic Sea marine mammals, describing their infection with some nematode species, evaluating changes in the parasite loads and composition in pinnipeds and cetaceans by increased grey seal populations and higher infection rates in cods and other potential hosts. Further emphasis is placed on the increasing *C. osculatum* population inside the Baltic Sea and possible new parasite invaders introduced through invasive fish species (gobies) or via regular summer visitors from warmer waters.

## Materials and Methods

### Sampling and Morphological Examination

Parasites were obtained from two dissections of marine mammals at the German Oceanographic Museum in Stralsund, Germany, during winter 2019. The collected animals stranded along the beaches of Mecklenburg-Western Pomerania (see Table 1), were stored at − 20 °C and defrosted for dissection, or dissected directly after recovery. Inner organs and structures, including i. a. heart, ear canals, lungs, intestine, stomach, liver, lymphatic system, mesenteries and muscles where examined for nematodes. The museum is requested by the state of Mecklenburg-Western Pomerania to investigate stranded mammals after their death in order to analyse the cause of death and to subsequently protect the animal populations and prevent unnatural death, according to the contract LUNG 20.42/17.

**Table 1:** A list of the dissected marine mammals harbouring nematode parasites, including age, documented parasites, localities and possible cause of death

Animal Isolate no. (Species)	Age	Stranding locality and date	Parasite load/ species found	Site	Cause of death
B74/17 (*H. grypus*)	Ad	Rügen, 04.12.2017	Minor parasite load, *C. osculatum*	Stomach	Probably drowning (fishing gear)
B70/16 (*H. grypus*)	Juv	Usedom, 27.12.2016	Minor parasite load, *C. osculatum*	Stomach	Unknown
M147/18 (*H. grypus*)	Ad	Rügen, 16.10.2018	severe parasite load (192 g), *C. osculatum*	Stomach	Unknown
M159/18 (*H. grypus*)	Ad	Stralsund, 26.12.2018	Medium parasite load, *C. osculatum*	Stomach	Bacterial infection
M76/18 (*Ph. phocoena*)	Juv	Graal-Müritz, 06.07.2018	Minor parasite load, *Ps. inflexus*	Inner ear	Unknown
M154/18 (*Ph. phocoena*)	Ad	Nienhagen, 19.11.2018	Minor parasite load, *Ps. inflexus*, *S. minor*	Inner ear, Bronchia	Unknown

*Ad* adult, *C. Contracaecum*, g gramm, *H. Halichoerus*, *Juv* juvenile, *No* number, *Ph. Phocoena, Ps Pseudalius*, *S. Stenurus*, *Sub* subadult

Nematodes were found in the stomach of grey seals (*Contracaecum osculatum*) and in the bronchia (*Stenurus minor*) and inner ear (*Pseudalius inflexus*) of harbour porpoise. Parasites were cleaned from host tissue or host stomach contents and stored in 70% and 99% EtOH for further examination and analyses. In addition, biological parameters of the dissected hosts (see Table 1) and the location of the parasites were noted. The parasites were first identified by their morphology and location in the mammal, based on Cabrera [28] and Balbuena et al. [23]. Parasites were divided into anisakid and pseudalid nematodes. In the case of anisakids, all specimens in good condition could be identified belonging to the genus *Contracaecum* [29, 30].

### Molecular Analysis

DNA was extracted with Qiagen^®^ DNeasy Blood & Tissue Kit (Qiagen, Hilden, Germany) according to manufacturer’s protocol. The rDNA marker regions ITS-1, 5.8S, ITS-2 were amplified from a subsample of randomly chosen 45 anisakids (*Contracaecum* sp.) and parts of this region (only ITS-2) for 15 pseudalid nematodes, following Jovani and Tella [31]. PCR reaction for anisakid nematodes was performed using the primers TK1 (5′-GGCAAA AGT CGT AAC AAG CT-3′) and NC2 (5′-TTA GTTTCT TTT CCT CCG CT-3′) [32] (primer by TIB MOLBIOL GmbH Berlin). The PCR started with an initial step at 95 °C for 1 min, followed by 40 cycles of denaturation at 94 °C for 45 s, annealing at 55 °C for 45 s and elongation at 72 °C for 45 s, with a final step of 10 min at 72 °C. For pseudalid nematodes, tailor-made primers (here called primer 1 & 2), as described in [21], were used to amplify the ITS-2 region. The PCR started with an initial step at 94 °C for 3 min, followed by 39 cycles of denaturation at 94 °C for 1 min, annealing at 60 °C for 1 min and elongation at 72 °C for 1 min. The PCR cycle ended with 5 min at 72 °C. Primer concentrations were 10 µM.

For electrophoresis, 1% agarose in TAE 1X-buffer gel was used. Five µl of DNA PCR products were mixed with 1 µl of GelPilot^®^ DNA Loading Dye (Qiagen, Hilden, Germany), premixed according to the manufacturer’s protocol, and loaded for each sample. For orientation, 6 µl of GelPilot^®^ 1 kb Ladder (100) (Qiagen, Hilden, Germany), premixed according to manufacturer’s protocol, were added to the first pocket of the gel. Samples and ladder were dyed with GelRed^®^ Nucleic Acid Gel Stain (Biotium, Inc. Fremont, California), according to the manufacturer’s protocol. Electrophoresis was run at 80 V, 130 mA and 50 W for one hour. The samples showing bands were purified with a Qiagen QIAqick^®^ PCR Purification Kit (Qiagen, Hilden, Germany) according to the manufacturer’s protocol. Sequencing was performed at Microsynth SEQLAB GmbH, Göttingen, Germany.

### Sequencing and Phylogenetic Analysis

Phylogenetic analyses of the DNA sequences were performed using MEGA X software [33]. Alignments and phylogenetic trees were based on the software-intern functions and features. The calculated best fitting models for phylogenetic trees were chosen by subjecting the aligned sequences to the *Best fitting Model* tool of Mega X. For the trees, Maximum Likelihood (ML) analyses were run, with a Kimura 2-parameter (K2) model for anisakid nematodes and a Tamura 3-parameter (T92) model for pseudalid nematodes.

## Results

### Stomach Contents

The porpoises did not harbour any nematodes in their stomach. The stomach of four out of six porpoises could be analysed for nematodes. In the other two specimens, tissue loss prevented the examination of the stomach. In the grey seals, all stomachs contained a varying amount of anisakid nematodes (*Contracaecum osculatum*). In two seals the stomachs contained, besides parasites, remains of prey items. Among the remains were both a vast amount of otolithes (from gobies, gadids, clupeids and cyprinids) and pharyngeal teeth of cyprinids as well as whole roaches (*Rutilus rutilus*) and round gobies (*Neogobius melanostomus*) in various stage of digestion.

### Prevalence and Location Of Parasites

**Fig. 1:**
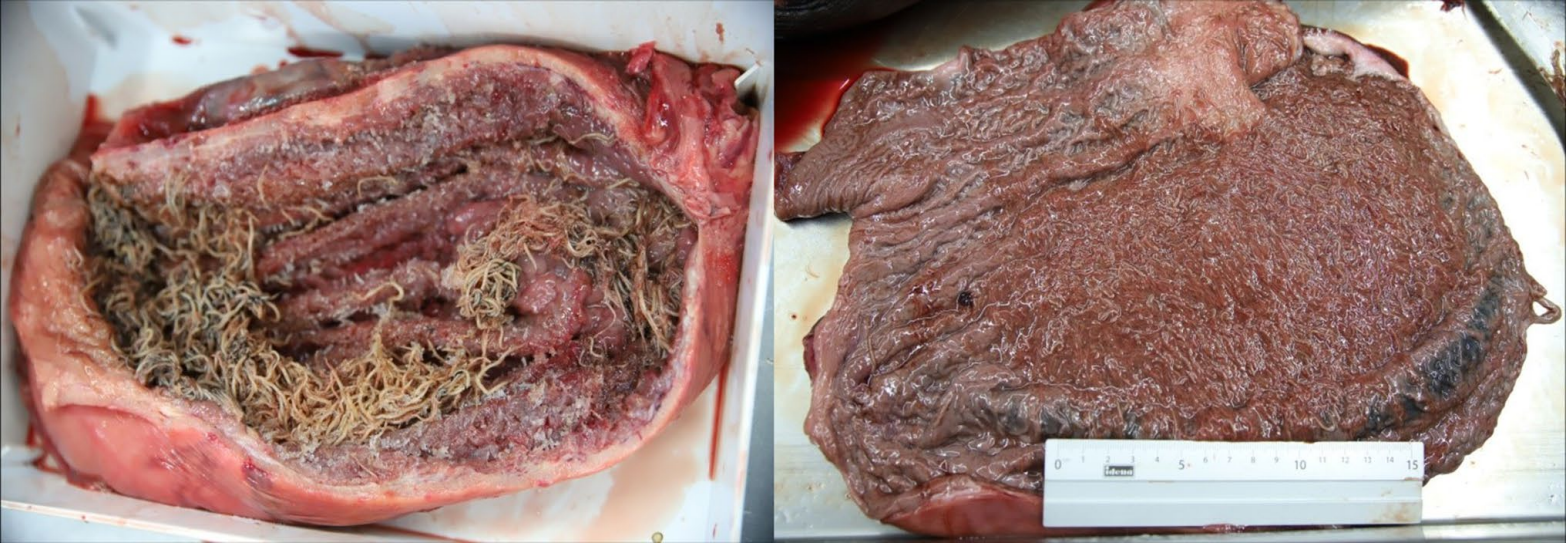
Opened stomachs of grey seals containing *Contracaecum osculatum.* Comparison of a medium infection (left) and a severe infection (right)

Of the four dissected grey seals and six porpoises, nematode prevalence was 100% for grey seals (*C.* osculatum) and 33.3% for harbour porpoise (combined for *Ps. inflexus* and *S. minor*). Nematodes were not counted, but the evaluation system of the German Oceanographic Museum (*none*, *minor, medium, severe*) was used to describe the parasite load instead for evaluating the intensity that ranged from minor to severe (see Table 1 and Fig. 1). Anisakid nematodes were isolated from the stomach of infected seals and in the oesophagus of a single heavily infected animal. Worms were not attached to the stomach walls, but scarification was observed. Pseudalid nematodes were located in the bronchia of the porpoises (*S. minor*), but also settled in the inner ear (*Ps. inflexus*), forming agglomerations.

### Molecular Identification

The nucleotide sequences from sanger sequencing of the isolated species were identified by comparing the obtained sequences to NCBI GenBank database. The received species were *Contracaecum osculatum* (rDNA marker regions ITS-1, 5.8S, ITS-2; *Identity* 99.42–100% and *Query* 98–100% to KU306623.1, [34], hosts M147/18 and M159/18, see Table 1) and two pseudalid nematodes *Pseudalius inflexus* (rDNA marker region ITS-2; *Identity* 96.17–100% and *Query* 99–100% to FJ767935.1 [21] host M154/18, see Table 1) and *Stenurus minor* (rDNA marker region ITS-2 and parts of it; *Identity* 98–100% and *Query* 93.86–100%, to FJ787302.1 [21] host M154/18, see Table 1). Sizes of the submitted nucleotide sequences ranged from 209 up to 948 base pairs (bp) in length. Differences in the length of sequences were detected between individuals of the same species, as in case of some pseudalid nematodes, only parts of the sequenced regions could be obtained. This was due to the quality of the sampled material. From the submitted 40 sequences with successful DNA extraction, 29 were anisakid and 11 pseudalid nematodes. Sequences were uploaded to GenBank, to extend the scope of the database in case of *Pseudalius inflexus* to five available deposits (MN475725–MN475728). Additional six sequences for *Stenurus minor* (MN491889–MN491894) and thirteen sequences for *Contracaecum osculatum* (MN428812–MN428824) from the Baltic Sea where deposited.

### Phylogenetic Analysis

**Fig. 2:**
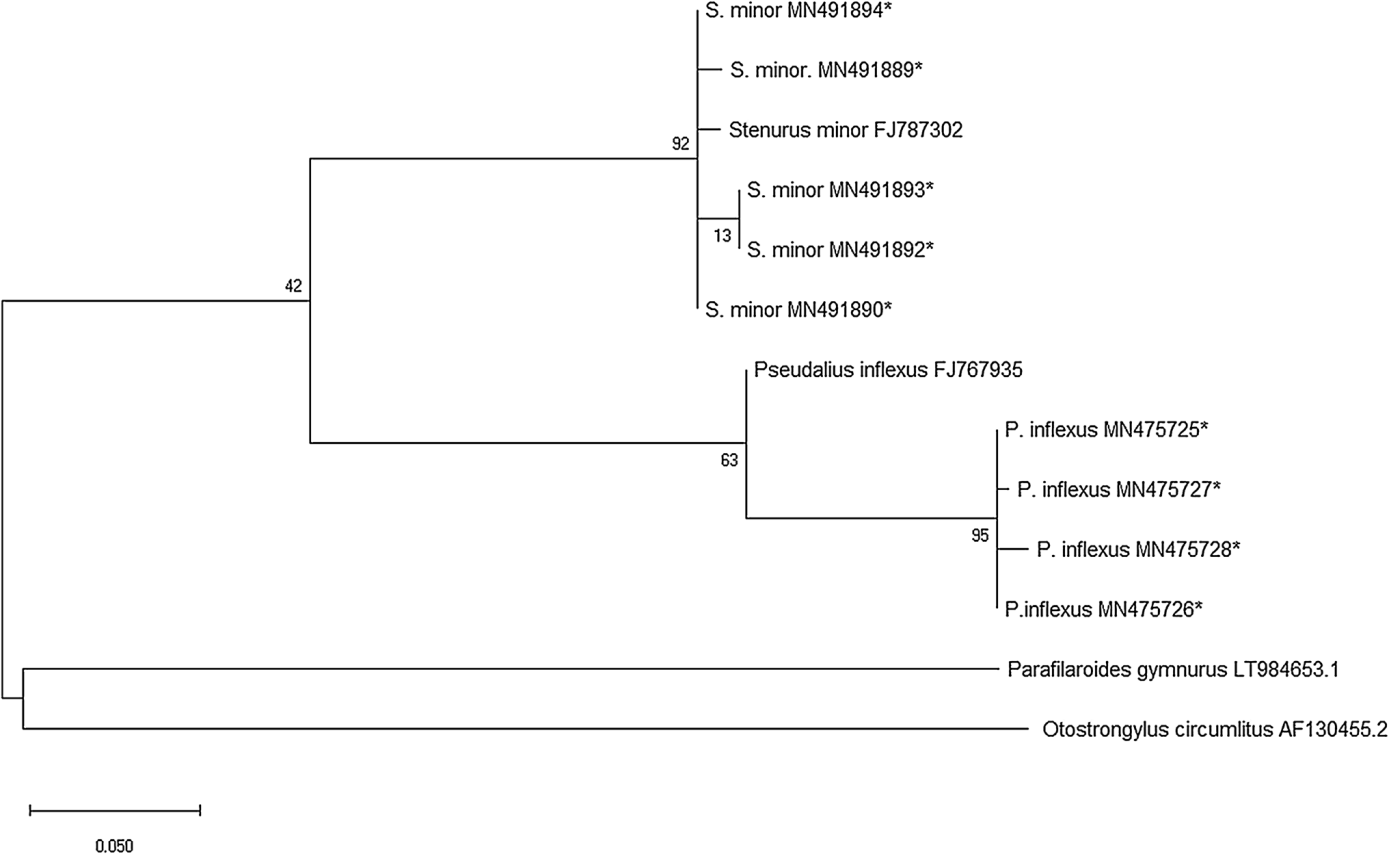
Phylogram of a Maximum Likelihood (ML) analysis of the ITS-2 marker dataset for pseudalid nematodes, comprising sequenced nematodes and selected references/outgroups (with GenBank accession numbers). A Tamura 3-parameter (T92) model is used and bootstrap values indicate confidence level in percent. Sequence length = 252 bp *Sequences created in this study

The phylogenetic trees created by ML-analysis (Maximum Likelihood) resulted in the obtained parasites grouping with the reference sequences from NCBI. Selected nematode species were used as (out-)groups (see Fig. 2, *Pseudalius inflexus* FJ767935; *Stenurus minor* FJ787302; *Otostrongylus circumlitus* AF130455.2, *Parafilaroides gymnurus* LT984653.1;). Size of the ITS-1, 5.8S, ITS-2 nucleotides reached up to 948 bp for *C. osculatum*, 509 bp for *Ps. inflexus* and 390 bp for *S. minor*. The pseudalid nematode phylogeny is shown in Fig. 2 building two differentiated clades with a strong bootstrap support of 92% for the clade of *S. minor* and the other clade build by *Ps. inflexus* sequences with a bootstrap value of 63%. Within the clade of by *Ps. inflexus*, the specimens from this study grouped with a very strong bootstrap support of 95%. The sequence MN491891 of *S. minor* was excluded from this comparison due to its short length. Obtained sequences of *C. osculatum* were uploaded to GenBank and showed a very high or complete similarity to the GenBank sequences AF411203 and KU306623.1 from *Contracaecum osculatum* found in *Halichoerus grypus* or *Gadus morhua* from the Baltic Sea (Bornholm Basin and Bothnian Bay). Sequence similarity ranged between 99.42 to 100%, while ten of the thirteen uploaded sequences were completely identical to the chosen references.

## Discussion

The present study provides an actual screening and molecular identification for the ongoing research on the nematode parasite fauna of marine mammals from the German Baltic coast of Mecklenburg-Western Pomerania. It identifies and confirms an anisakid nematode species infecting grey seals and two species of pseudalid nematodes infecting harbour porpoises. The parasite infection rates and the parasite loads (from minor to severe) were also considered. Possible new parasite species linked to invasive gobies and migratory fish from warmer waters could not be found.

### Parasites of Grey Seal

In the examined grey seals, the prevalence for anisakid nematodes was 100%, with infections ranging from minor to severe. The highest parasite load was found in two adult seals (with a biomass up to 192 g), which is consistent with previous findings [11, 15, 25]. In this study, however, only *Contracaecum osculatum* could be identified morphologically and molecularly in the seals. Predominance of this species over *Pseudoterranova decipiens* and *Anisakis simplex* was reported in the previously mentioned studies, but no sole infection with *C. osculatum* has been published so far. As only a subsample of the anisakid nematodes was analysed, the possible occurrence of another anisakid species cannot be excluded. However, a possible explanation for the identification of only *C. osculatum* in our subsample can be the actual high infection rates of the potential prey organisms of grey seals at the German Baltic coast with this nematode. Reports indicate that the re-population of the Baltic Sea with grey seals closed the life cycle of *C. osculatum* after a decade-long absence from the region. As stated by Palm and Bray (2014) [35] larval anisakid nematodes can survive at least three years in the teleost intermediate host and they may be carried to remote places. Both Haarder et al. [15] and Skrzypczak et al. [11] reported significantly higher prevalence and intensity of *C. osculatum* infections in Baltic Sea cods (*Gadus morhua*), increasing with fish size, contemporaneously to the increased grey seal population. As cod is an important prey item for grey seals in the Southern and Central Baltic Sea [9], infected cods will contribute to a higher parasite load in the infected seals. Other important prey items in the German Baltic Sea are herring (*Clupea harengus*) and gobies (Gobiidae), in particular the invasive round goby (*Neogobius melanostomus*) and black goby (*Gobius niger*), as well as roach (*Rutilus rutilus*) [7, 9]. The first two fish species are also known as paratenic hosts for *C. osculatum* in the Baltic Sea [36, 37], which may further increase the parasite load of grey seals. Lastly, *A. simplex* is only temporarily appearing with migrating herring and cannot close its life cycle in brackish water [25]. Unger et al. [36] demonstrated that this parasite is restricted to south-western parts of the Baltic Sea, where the Polish waters seem to be a distribution boundary, as migrating herring originating from the Kattegat and Skagerrak do not invade further into the Central Baltic Sea. *Pseudoterranova decipiens*, although increasing in abundance with recovering grey seal populations, is having much lower infection intensity than *C. osculatum*, and its range is also limited by low salt concentrations in the Eastern Baltic Sea and a strictly benthic life cycle [13, 34, 38]. These factors may as well contribute to a perceived sole infection with *C. osculatum* in the dissected grey seals in the present study.

### Parasites of Harbour Porpoise

The prevalence for the pseudalid nematode *Pseudalius inflexus* in the examined porpoises was 33.3%. A similar infection pattern for this parasite was reported from Balbuena et al. [23] for porpoises in Norwegian waters. Lehnert et al. [39] reported a far higher prevalence for pseudalid nematodes (90%) for porpoises of German and Norwegian Waters, but did not differentiate between the parasite species, as does Balbuena et al. [23] for the general prevalence of lung worm species sensu lato (98%). A mild co-infection of *Ps. inflexus* and *Stenurus minor* was only recorded for a single porpoise (16.6% prevalence) in this study, while the before mentioned studies describe pseudalid nematode co-infection as common (~ 50%) with higher prevalences for *S. minor* [22]. Such a prevalence and milder infection for the Baltic porpoises, compared to the Atlantic and North Sea, however, was also reported by Lehnert et al. [39]. The apparent absence of *Halocercus invaginatus* and *Torynurus convolutus* can be attributed to their small size and localisation in the lung parenchyma [40] and nasal cavity, as both tissues showed no signs of infection. Both species, however, have been found in North Sea and Baltic porpoises in previous studies [39, 40].

Anisakid and raphidascaridid nematodes were completely absent from the four digestive tracts of the investigated porpoises in this study. The prevalence of *A. simplex* for Baltic porpoises, although increasing with age and size, has been described ranging from 21% to 53% [25]. Lower prevalence has been reported from Lehnert et al. [35] (28%), Siebert et al. [20] (32%) and Herreras et al. [26] (40%). A possible explanation for the present results might be the state of decay and tissue lost by scavengers in some of the dissected animals or a diet shift by the porpoises. *Anisakis simplex* commonly uses euphausiacean crustaceans as an obligate first intermediate host. The function of teleost fish is limited to their role as a second intermediate, paratenic or accumulating host, while cetaceans are the definite host [25]. Since the salinity of the Baltic Sea is mostly too low for these crustaceans [41] and porpoises are rare in the Central Baltic Sea, the *Anisakis* larvae are most likely introduced through migrating herring from the North Sea [36] and later ingested by porpoises with their teleost intermediate hosts. Since herring only migrate into the Central Baltic Sea for spawning during spring and autumn [36, 42], it is the most likely explanation that all porpoises belonged to the two isolated populations of either the Baltic Proper or Danish waters and, at least at the time of their death or a certain time before, had no access to western herring and thus were *A. simplex* negative.

### Future Implications

We could demonstrate that the examined nematodes can be utilized as indicators for the population and the food spectrum of their mammalian final hosts. This research can be considered as an incentive to foster future research, reflecting the current state of knowledge and pointing out the need for further research on nematode parasites of Baltic marine mammals as final hosts.

Future studies on the anisakid nematodes of marine mammals in particular should use larger samples sizes to avoid possible overrepresentation of the predominant species. This might not be possible concerning availability of a higher number of stranded mammalian hosts, but the number of genetically analysed worms per host individual can be increased. Standardised samples should be taken from every infected individual to allow comparison between individual animals. Secondly, the actual sampling should focus on possible seasonal differences in parasite load, identifying whether seasonal changes in prey availability influence the real parasite load of the marine mammals. Thirdly, the diet of grey seals is quite variable and opportunistic and changes with availability and age. Invasive round gobies, an important part of the local seals diet, are now colonising large parts of the Baltic Sea. The present study revealed them as prey, as well as fresh water cyprinids, suggesting hunting activities in the coastal inner bays (e.g. Darss-Zingst Peninsula/Bodden Chain), a shallow lagoon-like estuary with freshwater characteristics and less salinity compared to the brackish Baltic Sea (see Reimer [43], Kemsis [44] and Layka [45] for information on the fish parasite fauna of the Bodden water bodies). Round gobies are vectors of *C. osculatum* larvae [37], and the parasite also infects cyprinids [46] commonly found in the inner bay (typically roach and bream). Additional research effort should be placed on the prevalence and parasite load of these potential hosts of *C. osculatum*, to evaluate their influence on the seals, especially on younger animals that hunt smaller fish like the gobies. This also refers to the spread and biohazard of the potentially zoonotic *C. osculatum* into fish species used for human consumption in the Bodden, a place where inshore commercial fisheries still exist in Germany. Finally, although the protocol by Lehnert et al. [21] was strictly followed for molecular analysis, the results were different than described in this reference and the obtained sequences, in particular of the forward primer, were too short to be used in phylogeny. Possible improvements could be archived by either revising the PCR-protocol or constructing new primers on the now known ITS-1, 5.8S, ITS-2 sequences of the pseudalid nematodes.
